# Human Face Recognition in Horses: Data in Favor of a Holistic Process

**DOI:** 10.3389/fpsyg.2020.575808

**Published:** 2020-09-15

**Authors:** Léa Lansade, Violaine Colson, Céline Parias, Fabrice Reigner, Aline Bertin, Ludovic Calandreau

**Affiliations:** ^1^PRC, INRAE, CNRS, IFCE, University Tours, Nouzilly, France; ^2^LPGP, INRAE, UR1037 Fish Physiology and Genomics, Rennes, France; ^3^Unité Expérimentale de Physiologie Animale de l’Orfrasière, Nouzilly, France

**Keywords:** face recognition, *Equus caballus*, animal cognition, human–animal relationship, horse model

## Abstract

Recent studies have demonstrated that horses can recognize humans based simply on visual information. However, none of these studies have investigated whether this involves the recognition of the face itself, or simply identifying people from non-complex external clues, such as hair color. To go beyond this we wanted to know whether certain features of the face were indispensable for this recognition (e.g., colors, hair or eyes). The 11 horses in this study had previously learned to identify four unfamiliar faces (portrait view and in color) presented repeatedly on a screen. We thus assessed whether they were able to identify these same faces spontaneously when they were presented in four other conditions: profile view, black and white, eyes hidden, changed hairstyle. The horses’ performances remained higher than chance level for all the conditions. In a choice test under real conditions, they then approached the people whose face they had learned more often than unknown people. In conclusion, when considering all the individuals studied, no single facial element that we tested appears to be essential for recognition, suggesting holistic processing in face recognition. That means horses do not base their recognition solely on an easy clue such as hair color. They can also link faces from photographs with people in real life, indicating that horses do not process images of faces as simple abstract shapes.

## Introduction

Facial recognition capacities have been increasingly studied in animals. They have focused on within- species recognition (pigeons: Nakamura et al., 2003; sheep: Ferreira et al., 2004; capuchins: Pokorny and de Waal, 2009; cattle: Coulon et al., 2011; macaques: Schell et al., 2011) and also of human faces (dogs: Huber et al., 2013; sheep: Knolle et al., 2017; dogs: Mongillo et al., 2017; bees: Avargues-Weber et al., 2018). Similarly, the horse appears to be able to recognize individuals and process faces. Studies have shown it capable of cross-modal recognition of its conspecifics (Proops et al., 2009). Horses can also express emotions through characteristic facial expressions (Dalla Costa et al., 2014; Gleerup et al., 2015; Hintze et al., 2016; Lansade et al., 2018; Trindade et al., 2020) and are able to differentiate these expressions (Wathan et al., 2016). Other studies have demonstrated that horses can identify human beings. For example, they can associate a voice with the sight of a specific person (Lampe and Andre, 2012; Proops and McComb, 2012). Similarly, they can link a facial expression depicting an emotion (joy or anger) with the corresponding vocalization (Nakamura et al., 2018; Trösch et al., 2019). Moreover, horses are capable of identifying in real life a person they had previously only seen in a photograph or video, and of adapting their behavior according to the facial expression or behavior that person had demonstrated (Smith et al., 2016; Proops et al., 2018; Trösch et al., 2020).

Two other studies focused on recognition of human faces. The first, conducted on four horses showed that they could learn to differentiate between two faces and then transfer that facial recognition during a field trial by passing more time with the person whose photograph had been associated with a reward (Stone, 2010). The second demonstrated that horses can both learn to differentiate faces of unknown people from a photograph and also spontaneously identify the photograph of a person they had encountered in real-life, despite not having seen that person for 6 months (Lansade et al., 2020). However, none of these studies has investigated whether this involves the recognition of the face itself, or simply identifying people from non-complex external clues, such as hair color.

This study thus aimed to determine whether horses based their recognition on a single, salient element, possibly even external to the face such as the hairstyle, or on more holistic face processing.

We tested horses which had previously been trained to identify four faces (the “recurrent faces”) from photographs presented repeatedly (portrait views, in color). During the tests, we presented the horses with the same recurrent faces under four different conditions: profile, in black and white, with eyes hidden and changed hairstyles. We chose to mask the eyes and the hair with accessories (sunglasses and a wig) rather than masking them a posteriori, so that the portraits remained more realistic, and were less disturbing for the animals. We hypothesized that if horses’ recognition ability was based only on a single clue (e.g., eyes) their performances would not differ from chance level under the conditions in which this clue was modified. Finally, to ensure that horses made the link between faces learned on a screen and people in real life and thus did not process facial images as simple abstract shapes, we conducted a choice test with real people (people whose faces had been presented repeatedly vs. totally unknown people).

## Materials and Methods

### Subjects

The study was conducted on 11 3-year-old female Welsh breed horses, bred at the Animal Physiology Experimental Unit PAO, INRA doi: 10.15454/1.5573896321728955E12). They were kept in a group at pasture or in a large stall with straw bedding. They had free access to fodder and water. From birth, they had been handled daily by a team of around 15 people (mucking out the stables, distributing food, walking in hand and carrying out basic health care such as clipping hooves, vaccinations, etc.).

### Set Up and Previous Training to Touch the Recurrent Faces

**FIGURE 1:**
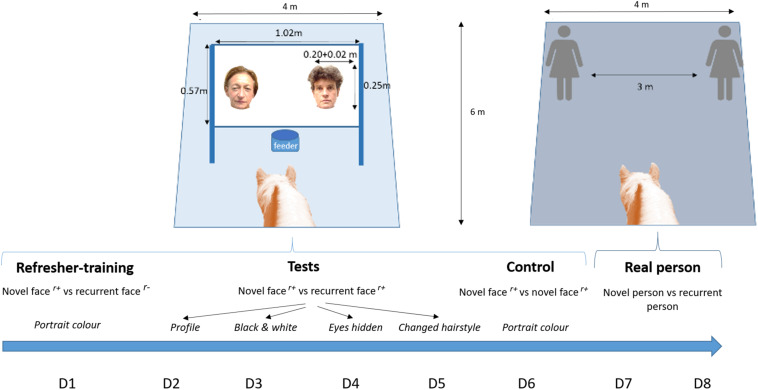
Experimental set up. r^+^, reward (pellets); r^–^, no reward; D, day.

The animals were tested individually in a testing area (6 m × 4 m) equipped with a video camera (Figure 1). A system consisting of a tactile screen (1.02 m × 0.57 m) linked to a computer with an automatic pellet distributor was located at the end of the area. Horses were led into the area and let loose in front of the system (see Lansade et al., 2020). For the previous training, and the sessions performed for this experiment, horses were individually tested in daily sessions of 32 trials, conducted between 9:00 and 17:00. Each trial began with a blank screen. After 30 s, two photographs appeared simultaneously on the screen. When the horses touched one of them with their nose or did nothing for 30 s the screen became blank again. A reward (5 g of pellets) fell automatically into a feeding trough located just below the screen, according to the conditions described below (training trials or test trials).

Prior to the present experiment, the horses had been trained to touch a “recurrent face” (among 4, Caucasian women, all unknown to the horse in real life) presented on a screen opposite a systematically different novel face to obtain pellets. The four recurrent faces became familiar over successive trials. Each of these “recurrent faces” was presented eight times per training session in a semi-randomized order (one “recurrent face” was never presented more than twice consecutively). Several intermediary steps were conducted to reach this objective (initially, one of the four recurrent faces was presented opposite a black circle, and then opposite novel objects and finally opposite novel faces). The horses learned to complete each step successfully (75% of correct responses on two consecutive sessions) within 2–5 sessions respectively, see Lansade et al. (2020) for more details.

The images of the recurrent and control faces were digital photographs taken by ourselves with a NIKON D3300. The photographs of novel faces were obtained from the Internet (192 novel faces were used for the present experiment from the refresher training to the tests: one per trial, and there were 6 sessions of 32 trials). The faces were all of adult women. All the images were edited using ImageJ software. The images were cropped and the background was white. Brightness and contrast were automatically adjusted to control for differences in lighting conditions. Life-size photographs displayed on the screen were: 25 cm high and 20 +2 cm wide.

### Procedure

#### Refresher-Training *– Recurrent Face vs. Novel Portrait Face Presented in Color*

The day before the tests, we checked that all animals reached at least 75% of correct responses (touching one of the four recurrent faces when presented opposite a novel face) over a session of 32 training trials. The procedure was exactly the same as that used in the last step of the training process described above. Horses were rewarded only when they touched the recurrent face.

#### Tests – *Recurrent Face vs. Novel Face, Presented Under Four Conditions*

Over the 32 trials in one session, 24 were the same as those described for the refresher-training (each recurrent face was presented the same number of times *-i.e., six times-* in a semi randomized order). The other eight (test trials) consisted of presenting the photograph of a novel face opposite one of the recurrent faces, but taken under four different conditions (one condition per session, one session per day): profile, black and white, with large dark sunglasses hiding the eyes and with a wig changing the hairstyle (Figure 1). For the wig, if the person had brown hair, we chose a blond wig, and if she had short hair, we chose a long wig (and vice versa). For each test trial, both faces (novel and recurrent) were presented under the same condition (e.g., both wore the same glasses or had the same hairstyle). These test trials were interspersed among the training trials in a semi-random fashion (never more than twice consecutively and balancing presentation of the four recurrent faces between the left and right side of the screen). During the test trial, the four recurrent faces were presented the same number of times (i.e., twice for each condition). Importantly, during the training trials only the recurrent face was rewarded, whereas during the test trials rewards were given for both images (recurrent and novel faces), the aim of the tests being to determine the horse’s spontaneous choice without creating a learning bias to touch the recurrent rather than the novel face. This reward system is classically used in the literature (e.g., Pokorny and de Waal, 2009; Lansade et al., 2020), whereas the alternative, that is to say no reward would have led to a rapid extinction of the response.

#### Control - *Novel Face vs. Novel Face*

A control test in one session was conducted the day after the last test to check that the horses’ recognition of the recurrent faces was based on the familiarity of the recurrent face rather than on other clues (for details: Lansade et al., 2020). The procedure was identical to that described above for the tests, but this time, instead of the recurrent face, the face of a novel person was presented opposite another novel face, during the 8 probe trials, intespersed among 24 training trials.

#### Real Person – *Recurrent Person vs. Novel Person*

Each horse carried out four choice tests (two consecutive tests on two consecutive days). For each test, two experimenters were positioned in two opposite corners of the test area (Figure 1). One of them was the recurrent person whose photograph had been used as the recurrent face, the other a person unknown to the horses. The recurrent and the novel person were different for each test, so that eight different people (four recurrent people and four novel people) were mobilized for each horse. For a given horse, choice test 1 involved recurrent person 1 opposite novel person 1; choice test 2, involved recurrent person 2 opposite novel person 2, etc. The position and the order of presentation of the people were counterbalanced between the horses and the tests. An assistant released the horse into the area and waited outside. The experimenters then attracted the horse’s attention: they bent the upper part of their body forward while looking at the horse, stretching out their hands and rubbing the thumb and other fingers together every 5 s while making a “kissing” noise with their lips until the horse made a choice or for a maximum of 30 s. They previously practiced to synchronize their movements. Importantly, neither the assistant nor the experimenters were aware of the hypothesis being tested or whether they were a recurrent person or not. At the end of each test, the horse was led out of the test area by the assistant. For each of the tests, three responses were possible: the horse (1) did not touch anyone, (2) touched the recurrent person, or (3) touched the novel person.

### Data Analyses

Data were analyzed with XLSTAT software. The data from the refresher-training, the tests and the control were analyzed using Student’s *t*-tests to compare the overall performance of the 11 horses –i.e., the number of correct responses out of 32 trials (refresher-training) or 8 trials (tests and control) – to chance level (50%). A paired *t*-test was used to compare the performance between the four conditions. The data was analyzed only at the overall level and not at the individual level, due to the insufficient number of trials conducted per individual and per condition (only eight trials). Nevertheless, a descriptive analysis of the individual data is presented in the results.

In front of the screen, animals always made a choice. This is not the case for the choice tests with real people. Thus for these we considered only the tests during which the horse made a choice (horses made a choice in median[Q1;Q3]: 3.5[3;4] tests out of the 4 conducted). We then calculated the percentage of tests for which the horse approached the recurrent person out of the number of tests for which the horse made a choice. A Student’s *t*-test was used to compare this percentage to the chance level (50%).

A modified Bonferroni correction (Keppel, 1982) was applied to consider the 13 comparisons conducted in the whole study, which were above the significance level from 0.05 to 0.02 (*α_*MB*_ = df_*A*_ (α_*PC*_)/c* where *α_*MB*_ is the* Modified Bonferroni, *df*_*A*_ is the degrees of freedom, *α_*PC*_* is the usual alpha level (0.05) and *c* is the number of comparisons). *N* = 11, except for the “profile” condition during which a horse had to be led back to its stable due to noise outside the test area, and for the test with real people due to a horse requiring veterinary treatment following a minor injury (*N* = 10). The percentage of correct responses per condition and per animal are presented in Supplementary Table S1, with the variation coefficient of each variable.

## Results

### Refresher-Training

**FIGURE 2:**
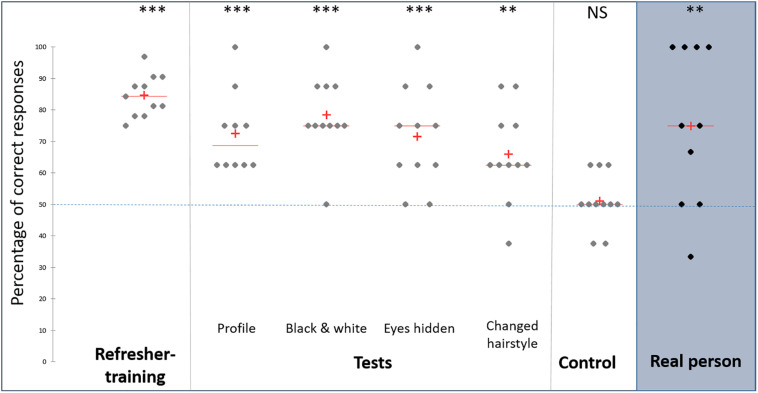
Percentage of correct responses. NS, non-significant; **p < 0.01; ***p < 0.001, Student’s *t*-test, calculated according to the level of chance (50%), *n* = 11 except for the profile condition and real person test (*n* = 10). ◆: individual, +: mean, ____: median, ————: level of chance.

Horses’ performances were significantly above chance during the refresher-training session (*t* = 17.35, *p* < 0.0001, *n* = 11, Figure 2).

### Tests

The performances during the tests remained significantly above chance whether the recurrent face was presented in profile (*t* = 5.51, *p* < 0.001, *n* = 10), in black and white (*t* = 7.47, *p* < 0.0001, *n* = 11), with eyes hidden (*t* = 4.50, *p* = 0.001, *n* = 11) or with changed hairstyle (*t* = 3.54; *p* = 0.005, *n* = 10, Figure 2). There was no difference in performance between the four conditions (Table 1). The descriptive analysis of the individual data in Supplementary Table S1 indicates that out of the 43 scores (11 animals ^∗^ 4 conditions, with one piece of data missing), 38 were above and only one below chance level. When focusing on individual by individual, 7/11 animals had scores systematically above chance whatever the condition, whereas only 1/11 had a score below chance, and in one condition only (animal number 5).

**TABLE 1:** Inter-conditions comparison.

	Black and white	Eyes hidden	Changed hairstyle
Profile	*t* = −1.24, *p* = 0.24	*t* = 0, *p* = 1	*t* = 1.26, *p* = 0.24
Black and white		*t* = 1.26, *p* = 0.24	*t* = 2.14, *p* = 0.06
Eyes hidden			*t* = 0.70, *p* = 0.50

*t-test for two samples.*

### Control

As expected, the performances during the control trial were not significantly different from chance level (*t* = 0.43; *p* = 0.68, *n* = 11, Figure 2).

### Real Person

Horses chose significantly more frequently the recurrent person than at a chance level (*t* = 3.18, *p* = 0.011, *n* = 10, Figure 2).

## Discussion

This study shows that, when considering all the horses together, face recognition performance remained significantly above chance level whatever the condition tested. The horses were also able to transfer face recognition learned on a screen to a test involving people in real life.

Before discussing the results further we will deal with a certain number of possible biases linked to this type of study. Firstly, as the horses were rewarded for whichever face they touched during the tests trials, we can exclude the effect of rapid learning of the recurrent face during the tests. This bias is discussed in Knolle’s study on sheep (Knolle et al., 2017). Another possible bias concerned the fact that we took the images of the recurrent faces while the novel faces came from the internet. Horses could have based their choices on similarities or differences between these two categories, even though all the images had been controlled in terms of contrast and light intensity. In the control test, using a photograph that had been taken with the same camera and under the same light intensity as the recurrent faces, the horses detected it simply as a photograph of an unknown person. This suggests that they did not base their recognition on how the image had been taken and confirms previous findings in another control test (Lansade et al., 2020). The choice with real people also enabled this possibility to be definitively excluded. Finally, the control test also eliminated horse choice relying on the fact that one photograph appeared several times in a row in the same session. Having excluded these biases, we can now discuss the two main findings of this study.

The first notable result is that when considering all the animals together, the horses’ performances remained significantly above chance level whatever the condition tested. Under the profile condition, the horses demonstrated a transfer from a front portrait to the same face in profile. Nevertheless, we observed a decrease in performance, similar to that observed in humans (Bruce and Young, 1986) or sheep (Knolle et al., 2017) under the same conditions. Overall performances also remained above chance level for the black and white photographs of the faces, which is also consistent with findings in sheep (Knolle et al., 2017). Horses therefore do not base their recognition solely on an easy clue such as color. Other than color, another plausible hypothesis is that horses simply recognize people from their hairstyle. However, in the condition with the wigs, overall performances were also above chance level, indicating that for most of the horses recognition was not based only on easy external clues such as the hairstyle. Although hair remained an important factor in recognition because it was under this condition that performances were the lowest (although not significantly). It was also under this condition that one individual had the lowest score. This is consistent with the literature on humans: recognition of a novel face is associated with a significant increase in the time spent looking at the external features, such as hair (Logan et al., 2017). Finally, the eyes were not an indispensable element for recognition. Certain studies in humans have suggested that eyes are one of the most important features for facial recognition (Hjelmas and Wroldsen, 1999), while others have shown that they are less relevant, particularly for novel faces, because they are affected to a greater extent by facial dynamics (Logan et al., 2017). This appears to be the case in horses.

The data were not analyzed statistically at the individual level due to the low number of trials carried out per individual and per condition (only eight). This choice of few trials, which is common for this type of study (e.g., Knolle et al., 2017; Lansade et al., 2020) was made to limit any potential learning during the tests. However, a descriptive analysis of the individual data confirms that the animals did not base their choice on one and the same prominent clue to identify the recurrent faces. This analysis also shows that for some of the animals, certain features could be more important than others, but these vary according to the subject. This would suggest that there is a certain inter-individual variability in the way subjects process human faces. This could potentially be linked to differences in personality, which would be an interesting avenue to investigate given the possibilities that exist to test personality in horses (Lansade et al., 2016).

Overall, it can therefore be assumed that for the majority of animals facial recognition was holistic in nature rather than being based on one prominent clue, even though certain individuals may privilege a specific feature. Other further studies are required in order to deepen our understanding of holistic face processing in horses. For example, it would be interesting to test a composite or inversion face effect, as already investigated in humans (Nakabayashi and Liu, 2014; Murphy et al., 2017) or other animal species (Burke and Sulikowski, 2013). It would also be interesting to test the horses with the profile of faces with eyes hidden, because horses have laterally positioned eyes and so might pay more attention to a single eye than to two eyes. Finally, further research could investigate the recognition of known individuals in real life, rather than of unknown people as in the present experiment: in humans, external features (e.g., hair) could be the most important factor for facial recognition of strangers (as suggested here), but not for recognizing known faces (Meinhardt-Injac and Persike, 2009; Logan et al., 2017).

The second notable result is that the horses made the connection between the learned photographs and people during the test with a real person. This ability to transfer learning of a face in two dimensions to a test under real conditions (three dimensions) supports Stone’s findings in a study using a less complex paradigm, consisting of only two sets of photographs and testing only four horses (Stone, 2010). Through this validation, we demonstrate that the horses in our study did not simply learn to discriminate between two abstract images in two dimensions, but they probably processed the image as a human face. To obtain a greater understanding of how horses process facial images, electroencephalograms could be conducted while testing horses in this type of task, to determine the mechanisms involved and to compare them with those implemented in other common species such as humans, dogs and monkeys (Tsao et al., 2008; Dilks et al., 2015).

## Conclusion

There would not appear to be one specific element of the face on which all the horses based their facial recognition. Although external features, such as hairstyle, seem to be important in identifying a novel person, horses are capable of using other cues, in particular internal features of the face itself, suggesting holistic recognition of human faces in the majority of individuals. Results also suggests a certain inter-individual variability in how faces are processed which merits further investigation. Finally, horses make a clear link between faces learned on a screen and people in real life, which indicates that they do not process facial images as simple abstract shapes. This knowledge in a non-primate model contributes to improving understanding of phylogenetic evolution of facial recognition processes.

## Data Availability Statement

The raw data supporting the conclusions of this article will be made available by the authors, without undue reservation.

## Ethics Statement

The animal study was reviewed and approved by Le Comité d’Éthique en Expérimentation Animale Val de Loire (CEEA VdL), France, reference number: 19.

## Author Contributions

LL devised the study and developed the experimental procedure with VC, CP, and LC. LL, CP, and FR conducted the experiments. LL wrote and revised the manuscript with significant input from VC, AB, and LC. All the authors approved the final version of the manuscript and agreed to be held accountable for the content.

## Conflict of Interest

The authors declare that the research was conducted in the absence of any commercial or financial relationships that could be construed as a potential conflict of interest.
