# Supplementary material for: Human Face Recognition in Horses: Data in Favor of a Holistic Process
Source: Front Psychol. 2020 Sep 15;11:575808. doi: 10.3389/fpsyg.2020.575808 (PMC7522352; doi:10.3389/fpsyg.2020.575808)
Supplement: Supplementary file 1 [file Table_1.DOCX]

**Supplementary Table S1.** Percentage of correct responses per condition and per animal

*cv: coefficient of variation*

|  |  | | | |  |  |  |
| --- | --- | --- | --- | --- | --- | --- | --- |
| Animal | Training  *(32 trials)* | Profile  *(8 trials)* | Black & white  *(8 trials)* | Eyes hidden  *(8 trials)* | Changed hairstyle  *(8 trials)* | Control  *(8 trials)* | Real person  *(4 trials)* |
| 1 | 96.875 | 62.5 | 50 | 50 | 75 | 62.5 | no data |
| 2 | 90.625 | 62.5 | 75 | 50 | 62.5 | 50 | 100 |
| 3 | 78.125 | 62.5 | 100 | 75 | 62.5 | 37.5 | 100 |
| 4 | 84.375 | 75 | 87.5 | 75 | 50 | 50 | 100 |
| 5 | 87.5 | 87.5 | 75 | 87.5 | 37.5 | 50 | 75 |
| 6 | 81.25 | 100 | 87.5 | 62.5 | 87.5 | 62.5 | 50 |
| 7 | 78.125 | no data | 75 | 62.5 | 75 | 37.5 | 100 |
| 8 | 87.5 | 62.5 | 75 | 100 | 62.5 | 50 | 33.33 |
| 9 | 75 | 75 | 87.5 | 62.5 | 87.5 | 50 | 66.66 |
| 10 | 81.25 | 62.5 | 75 | 75 | 62.5 | 50 | 50 |
| 11 | 90.625 | 75 | 75 | 87.5 | 62.5 | 62.5 | 75 |
| *cv* | *0.075* | *0.169* | *0.153* | *0.212* | *0.215* | *0.163* | *0.314* |
